# Human Papillomavirus and Cervical Cancer^1^

**DOI:** 10.3201/eid1011.040623_09

**Published:** 2004-11

**Authors:** Elizabeth R. Unger, Eliav Barr

**Affiliations:** * Centers for Disease Control and Prevention, Atlanta, Georgia, USA;; † Merck Research Laboratories, West Point, Pennsylvania, USA

**Keywords:** HPV, cervical cancer, vaccine

Though cervical cancer is highly curable when detected early, it remains one of the leading causes of cancer death in women worldwide. Early detection is effective because the precursor lesions evolve slowly into invasive cancer, typically over a period of >10 years. These precursor lesions (dysplasias or cervical intraepithelial neoplasias [CIN]) are detected with cervical cytology screening, the Pap smear. In every country where a Pap smear screening program has been introduced, rates of cervical cancer have been substantially reduced. The discovery that human papillomaviruses (HPV) are etiologically linked with cervical cancer has led to efforts to apply this knowledge to improve cervical cancer screening and to potentially prevent cervical cancer through vaccination.

## HPV and Cervical Cancer

HPV is not a single virus but a family of closely related viruses, each designated as a type, numbered in order of discovery. Typing is based on nucleic acid sequencing. More than 100 HPV types are known to exist, and at least 30 can be detected in the anogenital tract. No simple in vitro culture methods are available for identifying it, and serologic testing is insensitive. Techniques for identifying the virus are based on nucleic acid detection, either direct hybridization or after amplification. HPV types associated with malignancies are referred to as high-risk types, and those associated with warts (condylomas) are rarely found in cancers and are called low-risk types.

Sexual transmission is the dominant mechanism for acquiring genital HPV. Infection is usually transient and not associated with symptoms. An estimated 80% of sexually active women have been exposed. Studies have detected HPV in >90% of cancers worldwide, and plausible biologic mechanisms can explain oncogenesis. The magnitude of the risk association between HPV and cervical cancer is greater than that for smoking and lung cancer. However, infection alone is insufficient to cause cancer, and additional factors are required for neoplasia.

## HPV Vaccination as a Prevention Strategy

One investigational quadrivalent vaccine includes types 6, 11, 16, and 18. HPV-16 and HPV-18 (high-risk types) are found in 25% of all CIN I lesions and 70% of CIN II/III and anogenital cancers. HPV-6 and HPV-11 (low-risk types) are found in 25% of CIN I lesions and 90% of anogenital warts. Therefore a prophylactic vaccine against these four types would substantially reduce HPV-related disease.

Vaccine candidates have been evaluated in animal models of papillomavirus infection. The L1 protein of HPV is the major capsid protein and self-assembles into viruslike particles (VLPs). Species-specific VLP vaccines provide protection against infection and disease. Protection was associated with the development of neutralizing antibodies. Serum from vaccinated animals conferred protection to unvaccinated animals.

The HPV-6, HPV-11, HPV-16, and HPV-18 L1 VLP vaccine is manufactured in *Saccharomyces cerevisiae* (yeast), and yeast-derived vaccines have been given to millions of children and adults. The vaccine includes amorphous aluminum hydroxyphosphate sulfate adjuvant and is given in a 0-, 2-, 6-month dosing scheme. Phase I trials (300 participants) were performed to establish immunogenicity and tolerability of a range of doses of monovalent HPV L1 vaccines. Phase II trials (3,500 participants) were performed to establish the immunogenicity and tolerability of a range of HPV L1 VLP vaccine dose formulations and provide preliminary proof of concept. Phase III trials (>20,000 participants) will determine the efficacy of the HPV L1 VLP vaccine by using prevention of type-related CIN I, genital warts, and CIN II/III as the endpoints.

The results of the phase II trial of the HPV-16 VLP vaccine have been recently published (*1*). The primary endpoint of this trial in 2,392 young women was persistent HPV-16 infection (detection in consecutive visits) and HPV-16–related CIN. In 16- to 23-year-old women who were HPV-16–naïve at baseline, the vaccine was 100% effective; HPV-16 and CIN were detected in 41 unvaccinated (placebo) women and in no vaccinated women. The vaccine was generally well tolerated, and no serious vaccine-related adverse events were seen.

The phase III efficacy trial addressing women 16–23 years is underway. Approximately 25,000 women in 33 countries and 100 sites have been enrolled. The evaluation includes Pap testing and HPV polymerase chain reaction at defined intervals. An adolescent program (for girls 9–15 years of age) is ongoing to demonstrate vaccine immunogenicity and tolerability in boys and girls. In addition, a study with Nordic Cancer Registries is planned for long-term (>10 years) follow-up postlicensure to determine duration of efficacy, long-term safety, and replacement of vaccine types with other HPVs. Phase III programs will definitively evaluate clinical and public health impact of the HPV vaccine in adolescents and adult women.
